# *In Vitro* Evolution of Antibodies Inspired by *In Vivo* Evolution

**DOI:** 10.3389/fimmu.2018.01391

**Published:** 2018-06-21

**Authors:** Helena Persson, Ufuk Kirik, Linnea Thörnqvist, Lennart Greiff, Fredrik Levander, Mats Ohlin

**Affiliations:** ^1^Drug Discovery and Development Platform, Science for Life Laboratory, Stockholm, Sweden; ^2^School of Engineering Sciences in Chemistry, Biotechnology and Health, Royal Institute of Technology, Stockholm, Sweden; ^3^Department of Immunotechnology, Lund University, Lund, Sweden; ^4^Department of Clinical Sciences, Lund University, Lund, Sweden; ^5^Department of Otorhinolaryngology, Head & Neck Surgery, Skåne University Hospital, Lund, Sweden; ^6^Human Antibody Therapeutics, Drug Discovery and Development Platform, Science for Life Laboratory, Lund University, Lund, Sweden

**Keywords:** affinity maturation, antibody, antibody therapeutics, developability, evolution, humanization, immunoglobulin germline gene, somatic hypermutation

## Abstract

*In vitro* generation of antibodies often requires variable domain sequence evolution to adapt the protein in terms of affinity, specificity, or developability. Such antibodies, including those that are of interest for clinical development, may have their origins in a diversity of immunoglobulin germline genes. Others and we have previously shown that antibodies of different origins tend to evolve along different, preferred trajectories. Apart from substitutions within the complementary determining regions, evolution may also, in a germline gene-origin-defined manner, be focused to residues in the framework regions, and even to residues within the protein core, in many instances at a substantial distance from the antibody’s antigen-binding site. Examples of such germline origin-defined patterns of evolution are described. We propose that germline gene-preferred substitution patterns offer attractive alternatives that should be considered in efforts to evolve antibodies intended for therapeutic use with respect to appropriate affinity, specificity, and product developability. We also hypothesize that such germline gene-origin-defined *in vitro* evolution hold potential to result in products with limited immunogenicity, as similarly evolved antibodies will be parts of conventional, *in vivo*-generated antibody responses and thus are likely to have been seen by the immune system in the past.

## The Need for Antibody Evolution

Biopharmaceutical drugs, in particular monoclonal antibodies, have transformed treatment of disease in recent years. Currently (December 2017), more than 50 antibodies are approved for therapeutic use in the USA and/or the EU, 10 of which received their first approval in 2017 (1). Furthermore, nine antibodies are under regulatory review by the European Medicines Agency or the US Food and Drug Administration, and many more are undergoing evaluation in late stage clinical trials (1, 2). The large revenue associated with sales of many antibodies in clinical use, and the immense clinical benefit of antibodies in the treatment of a diversity of diseases, have stimulated enormous efforts and approaches that aim to develop antibodies for use in the clinic (2). These approaches include, e.g., utilization of well-behaving antibody scaffolds upon which antibodies are built by genetic engineering (3, 4), development of transgenic animals with human immunoglobulin genes that allow development of human antibodies in an *in vivo* setting (5, 6), humanization of highly effective antibodies of non-human (typically murine) origin (7), and exploitation of human *in vivo* immune responses for identification of high quality antibodies with unique properties (8–10). Antibodies with multiple specificities [generated either through development of dual-specific binding sites (11) or by design of bispecific (or higher order) constructs (12)] may engage appropriate effector cells to target cells carrying particular antigens, or facilitate antibody binding specifically to cells that carry both antigens engaged by the antibody’s specificities. Antibodies can also be further developed by selection of appropriate constant domains (natural or those designed for function) and glycosylation patterns so as to assure that they display appropriate effector functions [e.g., mediated through FcR binding (including antibody-dependent cellular cytotoxicity) or complement activation] and biological half-life (mainly an effect of FcRn binding) suitable for the intended application. Importantly, processes for identification and development of antibodies with appropriate properties in terms of “Chemistry, Manufacturing, and Control” have also been developed (13, 14) to ensure that a potential drug candidate can actually be turned into a viable commercial product.

The biophysical properties of antibodies as well as the affinity and specificity of the binding site can be affected by diversification of the variable (V) domains and subsequent selection of variants that display appropriate properties. Such modulation of antibody properties is often achieved by random mutagenesis, directed mutagenesis primarily of residues in complementarity determining regions (CDRs) (based on the assumption that CDRs carry much of the specificity-determining diversity of human antibodies), DNA shuffling, or chain shuffling, and subsequent selection of variants with improved properties. Selection can be achieved using a diversity of technologies like those relying on display on phage, yeast, or bacteria and the processes can achieve substantial maturation of e.g. the affinity, which may translate into improved performance in a given therapeutic setting. Ribosomal display even carries an inherent ability to evolve genes during the selection procedure (15) thereby advancing a maturation process, as selection from an original library proceeds. Given the potential size of molecular diversity space, it is only possible to assess a very limited fraction of that space in any given experiment. It is thus important to limit diversity to that acceptable for appropriate protein folding, as only such diversity is likely to deliver a functional product and thereby an appropriate outcome of the evolution process. Furthermore, it is envisaged that minimal diversification away from human antibody sequences that commonly occur *in vivo* is preferred in order to minimize the likelihood that the protein will be immunogenic and able to induce an anti-drug immune response during clinical use.

## Big Data as a Source of Inspiration for Development of Molecular Evolution Strategies

Big datasets, in particular those that are the results of next-generation sequencing (NGS), now offer extensive, unprecedented insight into immune antibody diversity as it is represented in human subjects (16). High-throughput single cell approaches to identify paired antibody heavy (H) and light chain sequences used in combination with NGS, and occasionally in combination with mass spectrometry-based proteomics, offer an additional dimension to antibody investigation and discovery that aid our understanding of immune repertoires also at a global level (17, 18). We are confident that this kind of large-scale insight can be translated into a multitude of actionable strategies in the field of improved antibody engineering and evolution. Exploitation of such technologies have indeed already defined routes through which immune responses develop and evolve in response to natural infection and vaccination (19, 20). Such studies have now provided a driving force for novel vaccine development in the field of infectious diseases (21–23).

## Antibody Evolution Beyond Conventional CDR—General Considerations

**Figure 1:**
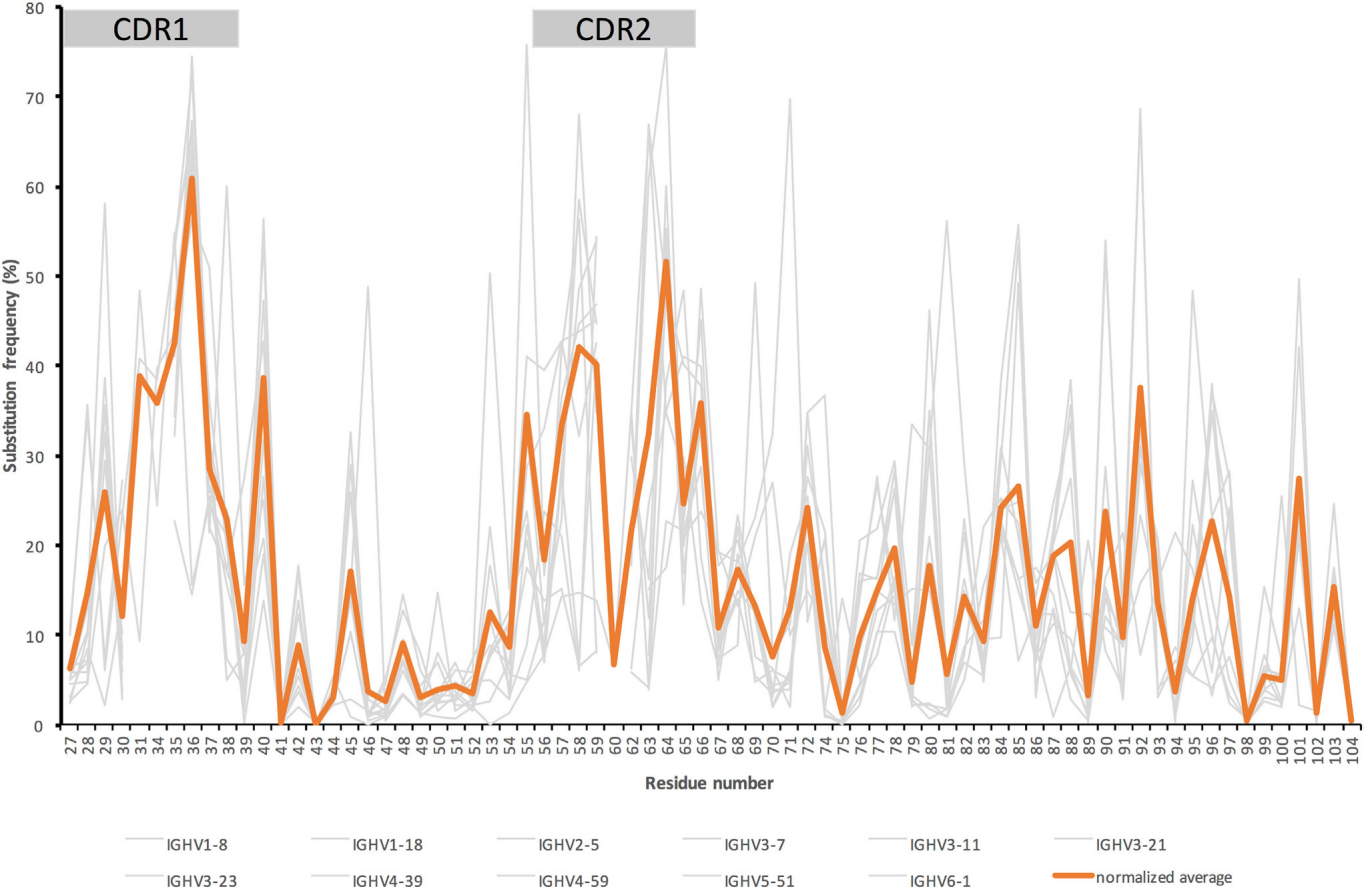
Substitution frequencies from the beginning of complementarity determining region (CDR)1 up to and including FR3 of the heavy chain of IgG encoded in bone marrow (39) by genes originally derived from 11 different germline genes with an origin in six different germline gene subgroups (gray) and the average substitution frequency (orange) seen in IgG derived from these germline genes [adjusted for the germline genes’ respective contribution to the overall antibody repertoire (40)]. [The illustration is a modified version of a figure published by us (38).] Residue numbering is according to the IMGT numbering system (41). This nomenclature defines CDR1 and CDR2 as residues 27–38 and 56–65, respectively.

**Figure 2:**
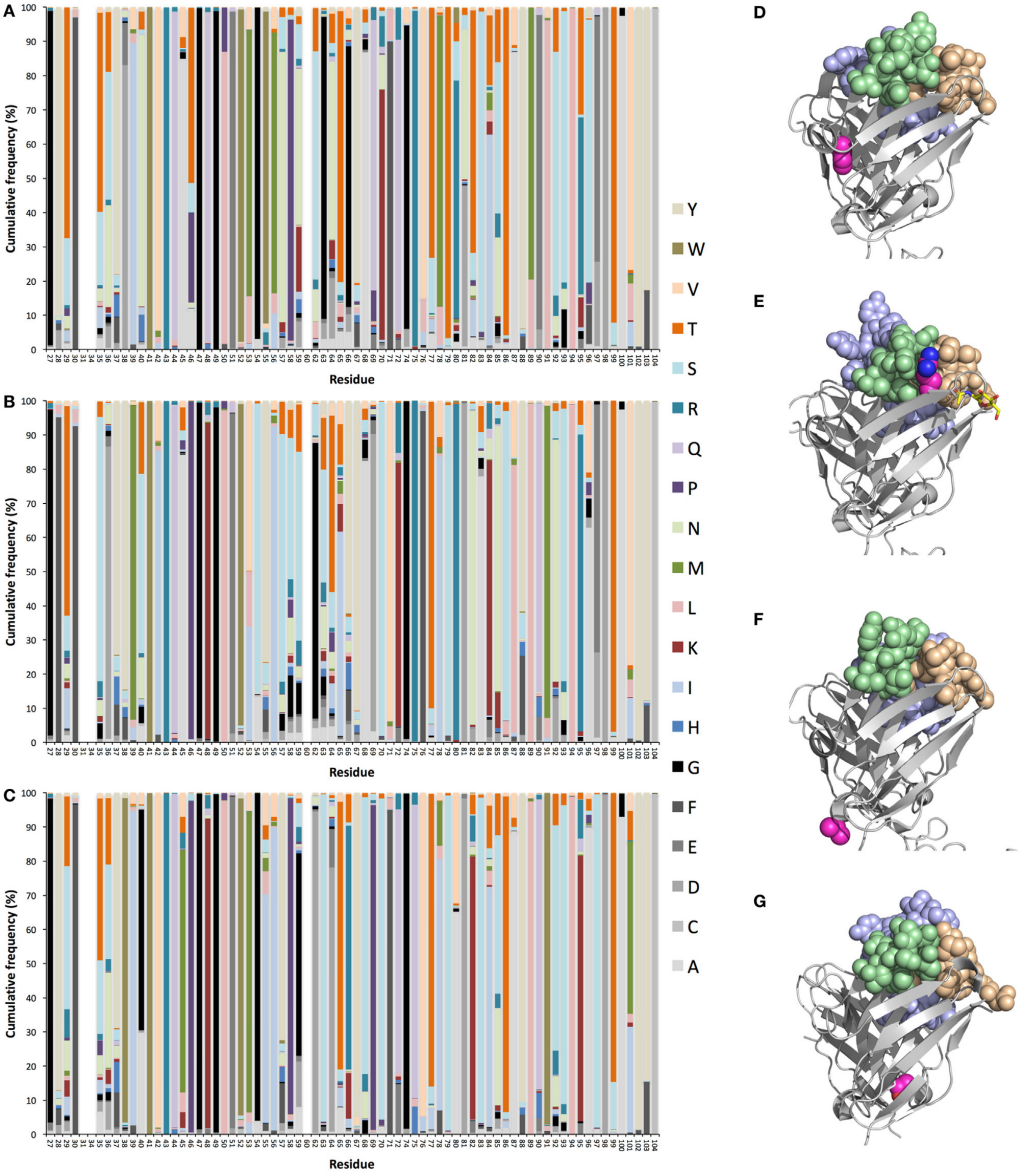
Diversification of heavy (H) chain variable (V) domain of IgG encoded in bone marrow (39) by genes originally derived from germline genes *IGHV1–8* **(A)**, *IGHV3–11* **(B)**, and *IGHV5–51* **(C)**, as previously described (38). Illustrations of such substitution patterns for an additional eight genes are available in Figure S1 in Supplementary Material. Structures of Fv of antibodies with H chain V domains with an origin in *IGHV1–18* (PDB: 3SDY) **(D)**, *IGHV1–8* (PDB: 3 × 3G) **(E)**, *IGHV3–11* (PDB: 4ZS6) **(F)**, and *IGHV5–51* (PDB: 4BUH) **(G)** illustrating examples of side chains of highly mutated positions [side chain atoms of which are shown in magenta (carbon), dark blue (nitrogen), and red (oxygen)]. The highlighted, highly mutated positions are position 71 of *IGHV1–18* **(D)**, position 80 of *IGHV1–8* **(E)**, position 96 of *IGHV3–11* **(F)**, and position 101 of *IGHV5–51* **(G)**. Atoms of H chain complementarity determining regions (CDRs) are illustrated in light brown (CDR1), light green (CDR2), and light blue (CDR3). All residue numbers are in accordance with the IMGT numbering rules (41). This nomenclature defines CDR1 and CDR2 as residues 27–38 and 56–65, respectively.

Importantly, large collections of diversified antibody sequences can also be used to inspire new thinking in the field of antibody evolution, in order to develop processes that efficiently evolve antibodies into variants that display appropriate binding properties and high developability. It was early on realized that antibody diversity at a global scale is focused into regions, the so-called CDRs, while intervening framework regions are more similar in sequence between different antibodies (24), in particular among sequences that belong to one and the same immunoglobulin clan (25). Such diversity is generated through variability in immunoglobulin germline-encoded genes, by the gene rearrangement process (focusing additional diversity into the third CDR), and by somatic hypermutation that may target mutational hotspots and substitution-prone codons that are focused to the parts of the genes that encode CDRs (26–29). These CDRs are well recognized to establish multiple direct interactions with antigen, although some interactions may certainly occur outside of these regions as well (25, 30, 31). Indeed, other parts of the immunoglobulin V domains have also been associated with functional antibody evolution. Studies of antibody *in vitro* evolution have suggested that “*mutations leading to higher affinity correspond to residues distant from the binding site*” (32) and that “*affinity maturation of antibodies with affinity in the low nanomolar range occurs most effectively via changes in “vernier” or second-sphere residues rather than contact residues*” (33). Furthermore, studies of antibody evolution *in vivo* suggested that “*There is a clear preference for mutations at the Ag-binding site. However, positions outside this region that also affect binding are often preferred targets for somatic hypermutations*” (34), but also that “*mutations in the contacting residues have an adverse effect on the antigen–antibody interaction*” (35), and that “*FWR mutations in noncontact residues are essential for the binding, breadth, and potency of most broadly neutralizing anti-HIV-1 antibodies*” (36). Although diversity in antibodies, generated at the level of diversity of germline genes themselves and through the hypermutation process, at a global scale focus onto the CDRs (Figure 1), others (37) and we (38) have demonstrated that diversity-generating hypermutation extends far beyond traditional CDRs in a manner that is defined by the germline gene origin of the antibody-encoding gene. Multiple sites, targeted by diversification in products of individual germline genes, can be identified in antibody H chains (Figures 2A–C; Figure S1 in Supplementary Material) (37, 38) and light chains (37). It thus appears that antibodies *in vivo* evolve through unique paths. These paths may be regulated by features built into the gene sequence itself (mutational hot- and cold-spots) or into features of their encoded proteins that favors evolution along particular trajectories. Irrespective of the specific reasons for the preferred paths of evolution, it is obvious that the immune system as such will have experienced and tolerated a substantial level of sequence diversity in antibodies, both within the CDRs and well beyond. This opens a window of opportunity for antibody *in vitro* optimization, the products of which, we hypothesize, may be well tolerated from an immunogenicity perspective in a therapeutic setting as it is already exploited by humoral immunity.

## Antibody Evolution Even Beyond Conventional CDR—Specific Examples

The sites available for natural evolution reside throughout the V domain of the H chain. Some germline genes offer opportunities for amino acid substitution in core residues, in H and light chain V domain interphase residues and in surface residues situated close to or far away from the paratope itself (38). A fourth hypervariable loop (CDR4) (42) located within FR3, but in close proximity to CDR1 and CDR2 of the folded protein, a region that may directly contact antigen (30, 43), may be particularly attractive for this purpose in the case of antibodies derived from some germline genes, such as *IGHV1–8, IGHV1–18*, and *IGHV5–51* (38). We also recently demonstrated patterns of insertions and deletions of entire codons, implied in the diversification of antibodies 20 years ago (44–46), that specifically target CDRs (including CDR4) of antibodies encoded by some germline genes (38). As such, diversification may be an important contributor to the development of particularly effective immune responses *in vivo* (47) it should be considered for *in vitro* antibody evolution purposes, possibly allowing us to reach parts of structure and antigen-interaction space not effectively reached by antibodies in other ways. Many other residues beyond CDRs have also been identified to carry diversity in a germline gene-defined manner. For instance, residue 80, a member of the V domain’s upper core (48), considered to be important for the position and conformation of H chain CDR2 (49) and stability of the V domain (50), is extensively targeted by substitution in products of some but not all germline genes (Figure 2) (38). Intriguingly, arginine 80 encoded by *IGHV1–8* (Figures 2A,E), but not by *IGHV3–7, IGHV3–11, IGHV3–21*, or *IGHV3–23* (38), is, based on diversification of H chains *in vivo*, a suitable target for diversification. Indeed, multiple other residues within the upper core are targetable by substitution in a germline-defined manner (38), modifications that may fine-tune the paratope (48, 51) and thereby perfect the antigen-binding properties. Indeed, evolution that might affect the structure/flexibility of H chain V domain CDR3 (52), the loop created through the VDJ rearrangement process, provides interesting opportunities in terms of perfection of the paratope. For instance, we recently observed substantial germline-defined differences in the ability to incorporate the 3′-most nucleotide of the IGHV-encoding gene into the rearranged sequence (53, 54). Many IGHV genes’ 3′-end has a sequence that it, if fully incorporated into the final product, would encode an acidic residue in position 107 of the ascending strand of CDR3. These side chains display opportunities to establish polar interactions, for instance to the side chain of amino acid 40 (located immediately after CDR1), not available to residues (commonly glycine, alanine, or valine) that are incorporated in cases where the full length of the IGHV gene is not used (53, 54). Importantly, the germline-encoded side chain of residue 40 is in many instances able to participate in polar interactions with a polar residue at position 107 in the ascending strand of H chain CDR3, and it holds capacity to be diversified (Figure S2 in Supplementary Material) (38). We postulate that diversification of residue 40 may be attempted as yet another way to modulate the structure of the binding site itself even beyond the nature of the residues in the paratope itself. Altogether, there are multiple germline origin-defined opportunities for paratope evolution in antibodies that may be exploited to achieve products with sequence similarity to antibodies that typically occur in human subjects.

Antibodies may need optimization for utilization in specific applications for reasons other than specificity and/or affinity. Developability of a candidate molecule is such an aspect that may require molecular optimization to provide a final candidate with properties like the absence of off-target binding, efficiency of manufacture, high stability, and ability to be incorporated in a suitable, formulated product. Indeed, antibodies, even those approved or in late stage clinical development, may differ widely in such respects (14). For instance, some side chains are sensitive to, e.g., oxidation, β-elimination, deamidation, or isomerization, and the protein may be poorly soluble, or sensitive to proteolysis and aggregation, processes that reduce the stability/homogeneity of the protein product. When such problems are identified in a lead antibody that is undergoing clinical development it may be necessary to optimize the protein sequence through molecular evolution (55), potentially through guidance by computational predictions (56), to keep these undesired processes at an acceptable level. It has been suggested that antibodies developed by phage display technology may be more prone to developability issues in relation to product specificity or solubility than antibodies developed in mice (57, 58), suggesting that such *in vitro*-generated antibodies more frequently may require optimization in this respect. We again hypothesize that a preferred solution to the problem may be sought through diversification of the protein in accordance with the mutational profile of antibodies of the same germline gene origin. This may be feasible even when sensitive residues are found far from the antibody’s paratope. For instance, methionine 101, situated far from the antibody’s binding site, as encoded by germline gene *IGHV5–51*, is surface exposed and might be prone to oxidation. This particular residue is, however, frequently diversified in IgG derived from germline gene *IGHV5–51* (37, 38), in particular to isoleucine, threonine, or valine (Figures 2C,G). This fact provides reassurance that it is worthwhile to attempt a designed mutational strategy (incorporating commonly occurring residues in this particular position) to resolve stability problems associated with this particular residue. Other residues, far from the binding site, such as residue 71 of *IGHV1–18* (Figure 2D) [in close proximity to the structure pattern-defining residue at position 76 (59)], and residue 96 of *IGHV3–11* (Figures 2B,F) are prone to substitution *in vivo*. A range of such sites have been defined by others, in the form of gene-specific substitution profiles (GSSP) (37), and us (38) (Figure 1; Figure S1 in Supplementary Material). Interestingly, a past study suggested that substitutions far from the binding site indeed stabilized antibodies that also carried destabilizing mutations in their paratope (60). However, although the authors were confident about that main conclusion of this study, it was retracted based on a lack of confidence in a subset of confirmatory data (61). Formal demonstration of the usefulness of targeting of paratope-distant residues in antibody stabilization is thus not fully in place. Nevertheless, these natural paths of evolution, in our opinion, forms a natural basis for antibody perfection with maximum chance of tolerability and minimal risk of induction of anti-drug antibody responses following treatment as such antibody sequences would have been commonly seen by the immune system in the past. This will be the case as long as the treated subject, given the substantial diversity of the immunoglobulin loci (40, 62, 63), actually encodes antibodies derived from the germline gene/allele in question.

## Conclusion

In summary, others and we have demonstrated substantial germline origin-unique substitution patterns in antibody V domains. Such patterns, even those beyond the classical CDRs, ought to be exploited in efforts aimed at affinity or specificity maturation, or optimization in terms of product developability. Future in-depth analysis of the outcome in terms of immunogenicity of antibodies perfected in this knowledge-driven manner will be required. However, we envisage that this procedure has true potential to establish antibodies with very limited immunogenicity as subjects treated with them are likely to have seen highly similarly, molecularly evolved, antibodies in the past as part of their own immune responses.

## Ethics Statement

This study was carried out in accordance with the recommendations of Regionala etikprövningsnämnden (Lund) with written informed consent from all subjects. All subjects gave written informed consent in accordance with the Declaration of Helsinki. The protocol was approved by the Regionala etikprövningsnämnden (Lund).

## Author Contributions

HP: conceived the study and manuscript preparation. UK and LT: bioinformatics pipeline development, bioinformatics analysis, and manuscript preparation. FL: initial bioinformatics pipeline development and manuscript preparation. LG: patient management and manuscript preparation. MO: conceived the study, bioinformatics analysis, and main responsibility for manuscript preparation. All authors approved the final manuscript.

## Conflict of Interest Statement

The authors declare that the research was con-ducted in the absence of any commercial or financial relationships that could be construed as a potential conflict of interest.

## Abbreviations

CDR, complementarity determining region; FR, framework region; H, heavy; NGS, next-generation sequencing; V, variable.
