# Supplementary material for: In Vitro Evolution of Antibodies Inspired by In Vivo Evolution
Source: Front Immunol. 2018 Jun 21;9:1391. doi: 10.3389/fimmu.2018.01391 (PMC6021498; doi:10.3389/fimmu.2018.01391)
Supplement: Supplementary file 1 [file Data_Sheet_1.PDF]

SUPPLEMENTARY INFORMATION

Germline gene-centric approach to *in vitro* evolution of antibody heavy chain variable domains inspired by *in vivo* antibody evolution

Helena Persson Lotsholm[1, 2], Ufuk Kirik[3, †], Linnea Thörnqvist[3], Lennart Greiff[4, 5], Fredrik Levander[3] and Mats Ohlin[3, 6*]

[1] Science for Life Laboratory, Drug Discovery and Development Platform, Stockholm, Sweden

[2] School of Biotechnology, Royal Institute of Technology, Stockholm, Sweden

[3] Dept. of Immunotechnology, Lund University, Lund, Sweden

[4] Dept. of Clinical Sciences, Lund University, Lund, Sweden

[5] Department of Otorhinolaryngology, Head & Neck Surgery, Skåne University Hospital, Lund, Sweden.

[6] Science for Life Laboratory, Drug Discovery and Development Platform, Human Antibody Therapeutics, Lund University, Lund Sweden

* Corresponding author: Dr. Mats Ohlin, Dept. of Immunotechnology, Lund University, Medicon Village building 406, S-22381 Lund, Sweden. Telephone: +46-46-222 4322; e-mail: mats.ohlin@immun.lth.se

† Current affiliation: The Novo Nordisk Foundation, Center for Protein Research, University of Copenhagen, Denmark

Supplementary Materials and Methods

Sample Preparation and Sequencing

Peripheral blood and bone marrow samples from six donors were, as previously described (Levin et al., 2017), collected (after approval by the regional ethical review board at Lund University) and used to prepare libraries of transcripts encoding immunoglobulin H chain V domains. Briefly, mononuclear cells were isolated from the samples, and divided into duplicates from which RNA was purified. cDNA was produced and subsequently amplified using Biomed2 primers (van Dongen et al., 2003), which targeted sequences encoding the constant domain (isotype-specifically) and the framework 1 region of the H chains, respectively. Finally, barcodes were introduced and the products were sequenced at the National Genomics Infrastructure (SciLifeLab, Stockholm, Sweden). Paired-end MiSeq technology (Illumina, Inc. San Diego, CA, USA) were used, with a 2 x 300 bp setup (Levin et al., 2017).

Processing of Sequence Data

As earlier described (Levin et al., 2017), the sequence data was processed using pRESTO 0.4.4 (Vander Heiden et al., 2014) and subsequently IMGT HighV-QUEST (Alamyar et al., 2012). In short, obtained FASTAQ raw data (accessible in the European Nucleotide Archive, with accession number PRJEB18926) were filtered, trimmed, paired, assembled and split into FASTA files according to isotype. Any sequences lacking particular isotype-specific sequences were discarded (Levin et al., 2017) before submission to IMGT HighV-quest (Alamyar et al., 2012). Eleven commonly expressed germline genes (IGHV1-8, IGHV1-18, IGHV2-5, IGHV3-7, IGHV3-11, IGHV3-21, IGHV3-23, IGHV4-39, IGHV4-59, IGHV5-51 and IGHV6-1) were identified via germline gene inference using IgDiscover and IgM encoding transcripts, as previously described (Kirik et al., 2017). The IMGT HighV-quest output for IgG sequences in bone marrow, originating in any of those germline genes, were used for the analyses of this study.

Analysis of the H Chain V Domains of IgGs

Further analysis steps for data presented in Figure 1-2 and Supplementary Figure 1 were performed as described before (Kirik et al., 2017). For data presented in Supplementary Figure 2, an in-house Java program was used to evaluate the frequency of each amino acid at position 40, when the residue at position 107 was either glutamic/aspartic acid, glycine or any other amino acid. Sequences inferred as unproductive were excluded, and duplicate sequences were only evaluated once. Duplicate sequences were identified using pRESTO 0.5.4 (Vander Heiden et al., 2014) and the CollapseSeq tool (n=5).

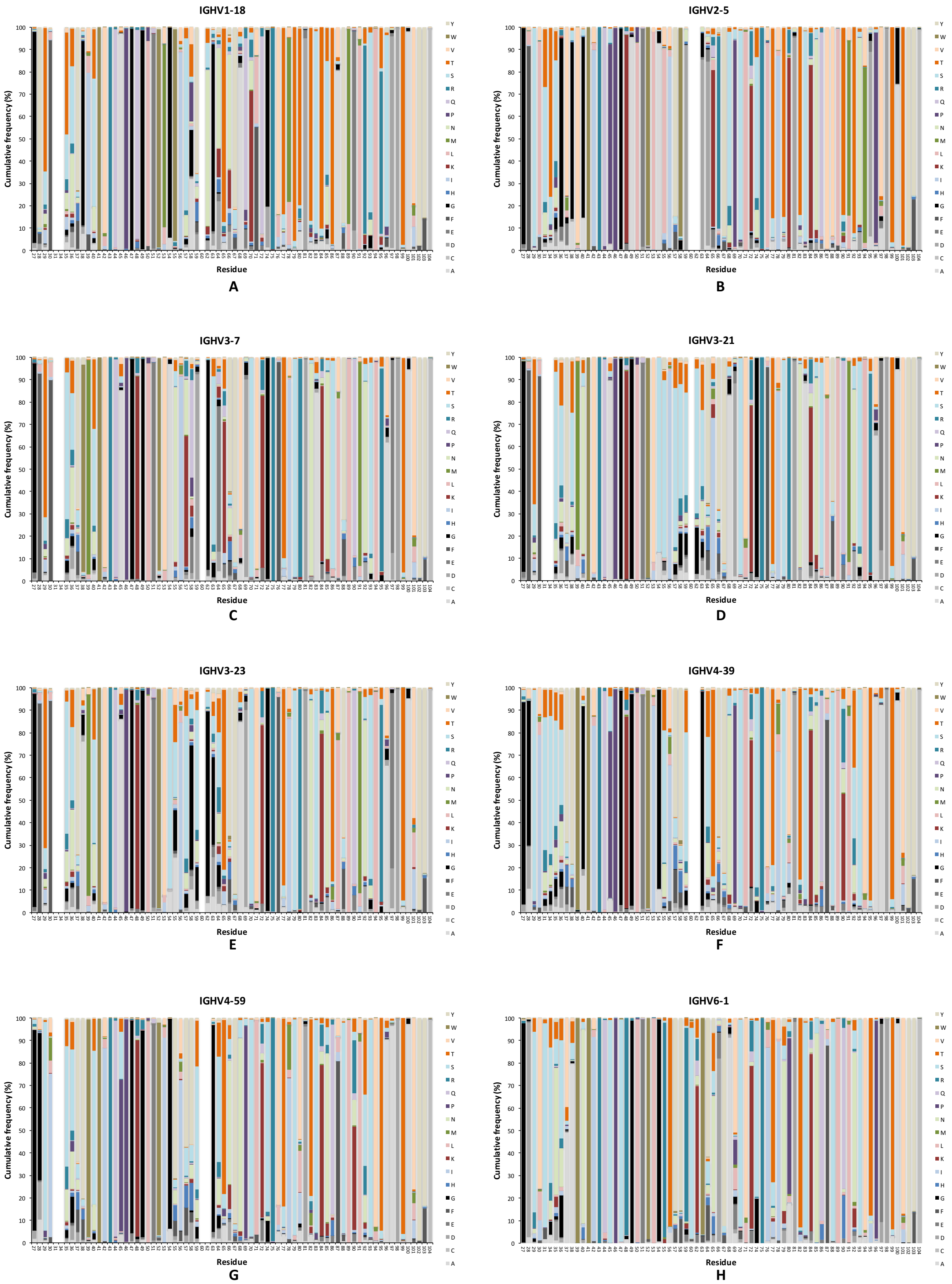

Supplementary Figure 1. Diversification of H chain V domain of IgG encoded in bone marrow by genes originally derived from germline genes *IGHV1-18* (**A**), *IGHV2-5* (**B**) *IGHV3-7* (**C**), *IGHV3-21* (**D**), *IGHV3-23* (**E**), *IGHV4-39* (**F**), *IGHV4-59* (**G**), and *IGHV6-1* (**H**), as previously described (Kirik et al., 2017). Illustrations of such substitution patterns for an additional 3 genes are available in Figure 1.

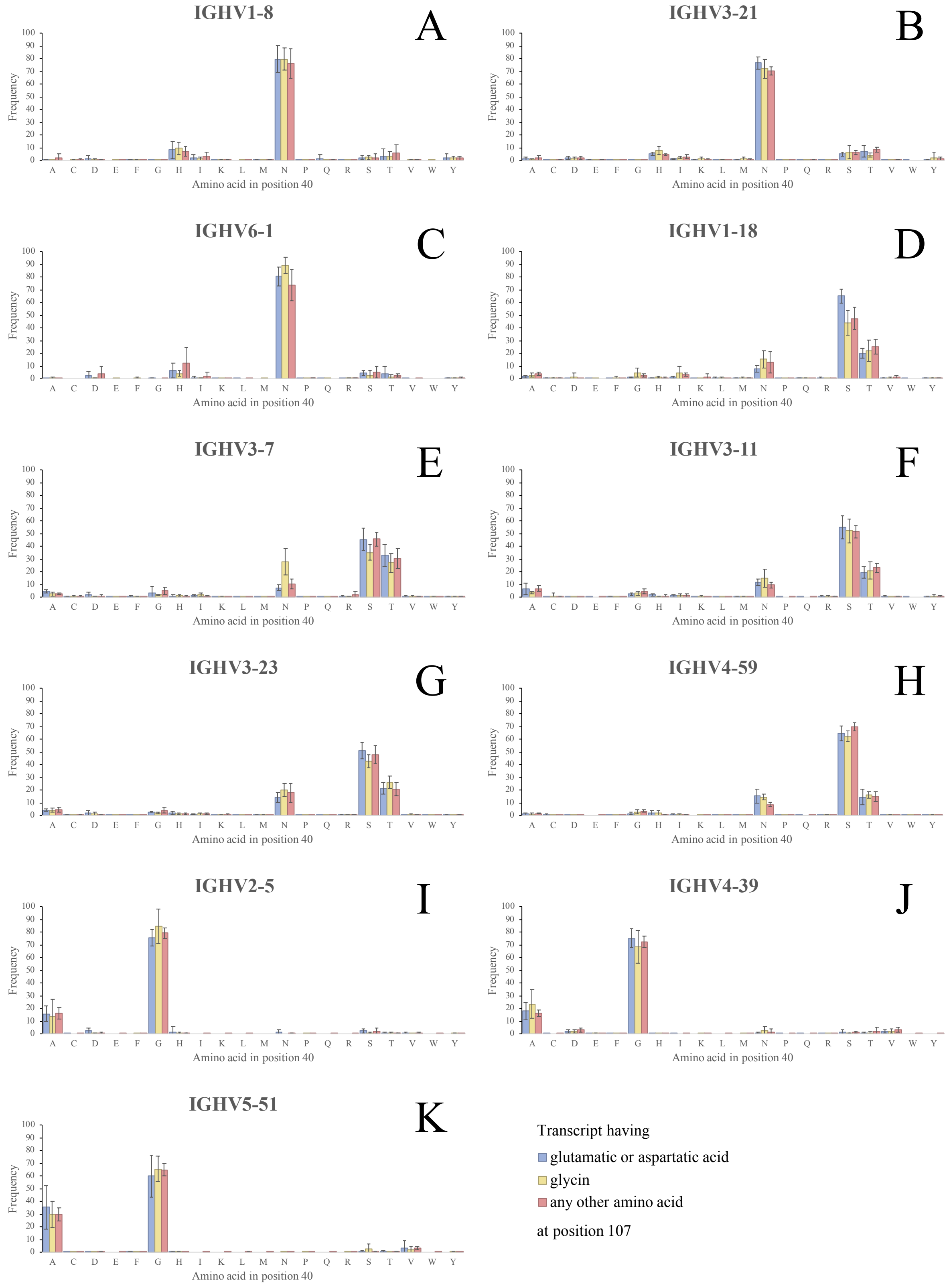

Supplementary Figure 2. Variability in H chain V domain residue 40 in IgG encoded in bone marrow (Levin et al., 2017) in relation to the residue at position 107 in sequences derived from 11 germline genes; *IGHV1-8* (**A**), *IGHV3-21* (**B**), *IGHV6-1* (**C**), *IGHV1-18* (**D**),

IGHV3-7 (**E**), *IGHV3-11* (**F**), *IGHV3-23* (**G**), *IGHV4-59* (**H**), *IGHV2-5* (**I**), *IGHV4-39* (**J**), and *IGHV5-51* (**K**). In germline configuration these genes encode asparagine (**A-C**), serine (**D-H**), and glycine (**I-K**) in residue 40 of the H chain V domain. Analysis was performed as outlined in Supplementary Materials and Methods.

References

Alamyar, E., Duroux, P., Lefranc, M.P., and Giudicelli, V. (2012). IMGT((R)) tools for the nucleotide analysis of immunoglobulin (IG) and T cell receptor (TR) V-(D)-J repertoires, polymorphisms, and IG mutations: IMGT/V-QUEST and IMGT/HighV-QUEST for NGS. *Methods Mol Biol* 882**,** 569-604. 10.1007/978-1-61779-842-9_32.

Kirik, U., Persson, H., Levander, F., Greiff, L., and Ohlin, M. (2017). Antibody heavy chain variable domains of different germline gene origins diversify through different paths. *Front Immunol* 8**,** 1433. 10.3389/fimmu.2017.01433.

Levin, M., Levander, F., Palmason, R., Greiff, L., and Ohlin, M. (2017). Antibody-encoding repertoires of bone marrow and peripheral blood-a focus on IgE. *J Allergy Clin Immunol* 139**,** 1026-1030. 10.1016/j.jaci.2016.06.040.

Van Dongen, J.J., Langerak, A.W., Bruggemann, M., Evans, P.A., Hummel, M., Lavender, F.L., Delabesse, E., Davi, F., Schuuring, E., Garcia-Sanz, R., Van Krieken, J.H., Droese, J., Gonzalez, D., Bastard, C., White, H.E., Spaargaren, M., Gonzalez, M., Parreira, A., Smith, J.L., Morgan, G.J., Kneba, M., and Macintyre, E.A. (2003). Design and standardization of PCR primers and protocols for detection of clonal immunoglobulin and T-cell receptor gene recombinations in suspect lymphoproliferations: report of the BIOMED-2 Concerted Action BMH4-CT98-3936. *Leukemia* 17**,** 2257-2317. 10.1038/sj.leu.2403202.

Vander Heiden, J.A., Yaari, G., Uduman, M., Stern, J.N., O'connor, K.C., Hafler, D.A., Vigneault, F., and Kleinstein, S.H. (2014). pRESTO: a toolkit for processing high-throughput sequencing raw reads of lymphocyte receptor repertoires. *Bioinformatics* 30**,** 1930-1932. 10.1093/bioinformatics/btu138.
